# Ameliorative Effects of *Lactobacillus paracasei* L14 on Oxidative Stress and Gut Microbiota in Type 2 Diabetes Mellitus Rats

**DOI:** 10.3390/antiox12081515

**Published:** 2023-07-28

**Authors:** Zhu Zeng, Yi Yang, Xinxin Zhong, Fangyin Dai, Shangwu Chen, Xiaoling Tong

**Affiliations:** 1 State Key Laboratory of Resource Insects, College of Sericulture, Textile and Biomass Sciences, Southwest University, Chongqing 400715, China; zhuzeng12@swu.edu.cn (Z.Z.); swu5997@email.swu.edu.cn (Y.Y.); fydai@swu.edu.cn (F.D.); 2 College of Food Science and Nutritional Engineering, China Agricultural University, Beijing 100083, China; swchen@cau.edu.cn

**Keywords:** probiotics, *Lactobacillus*, oxidative stress, type 2 diabetes mellitus, gut microbiota

## Abstract

Bioprospecting of more novel probiotic strains has attained continuous interest. This study aimed to investigate the beneficial effects of *Lactobacillus paracasei* strain L14, an isolate from a traditional Chinese dairy product, on type 2 diabetes mellitus (T2DM) rats. Preventive supplementation of strain L14 showed excellent anti-diabetic effects on high-fat diet/low-dose streptozotocin (HFD/STZ)-induced T2DM rats. It significantly reduced hyperglycemia, protected pancreatic β-cell and liver function, and ameliorated oxidative stress while considerably improving dyslipidemia and inflammation. Furthermore, the strain modulated the gut microbiota to alleviate gut dysbiosis. Interestingly, most of these biochemical parameters could even restore to normal levels by the intervention of strain L14. The whole-genome sequencing of L14 was performed to provide a critical molecular basis for its probiotic activities. Genes related to antioxidant systems and other beneficial microbial metabolites like exopolysaccharides (EPS) biosynthesis were found. This study demonstrates that probiotic *L. paracasei* L14 has good potential for applications in functional food and pharmaceutical industries.

## 1. Introduction

As a chronic metabolic disease, diabetes presents a significant risk to public health. T2DM accounts for approximately 90% of all cases. About 537 million people between the ages of 20 and 79 suffered from diabetes worldwide in 2021, which increased by 16% compared to 2019 and accounted for 10.5% of the global adult population. Furthermore, about half of diabetic cases remain undiagnosed [1]. Some of the primary characteristics of T2DM include hyperglycemia, insulin resistance (IR), and inadequate insulin production. T2DM is also related to oxidative stress, inflammatory status, dyslipidemia, and gut dysbiosis [2]. Studies have shown that gut microbiota contributes critically to the pathophysiology of T2DM via host–microbiota interactions, potentially by affecting gut barrier function, systemic inflammation, oxidative stress, gut hormone secretion, and energy metabolism [3,4].

Probiotics, especially *Lactobacillus* and *Bifidobacterium*, have emerged as a novel strategy to alleviate the occurrence and development of T2DM by regulating gut microbiota and producing advantageous metabolites like short-chain fatty acids (SCFAs) and other compounds [5]. For example, *L. paracasei* HII01 exhibited antihyperglycemic action in rats with T2DM via gut microbial regulation and promoting gut functionality [6]. *L. plantarum* SHY130 showed anti-diabetic properties in mice with HFD/STZ-induced T2DM by improving glucose homeostasis via the regulation of the enteroinsular axis [7]. Although extensive research has confirmed the beneficial effect of probiotics in preventing and treating T2DM, the specific regulatory mechanisms remain unclear. In addition, probiotics show strain-specific properties and different strains may work via different mechanisms. Therefore, screening and clinical verification of more specific probiotics for T2DM are necessary.

*L. paracasei* L14, isolated from a traditional Chinese dairy product, shows high dipeptidyl peptidase 4 (DPPIV) and α-glucosidase inhibitory activities in vitro [8,9]. This study focuses on the preventive effects of strain L14 on T2DM rats and its possible mechanisms.

## 2. Materials and Methods

### 2.1. The T2DM Rat Model Induced by HFD/STZ

The Vital River Laboratory Animal Technology Co., Ltd. (certificate number SCXK (Beijing) 2016-0001, Beijing, China) supplied the 18 male Sprague–Dawley (SD) rats (8 weeks old, specific-pathogen-free (SPF)), which were kept at Tsinghua University. The Tsinghua University Institutional Animal Care and Use Committee (SYXK2014-0024; Beijing, China) approved all the animal protocols used in this study. The Association for Assessment and Accreditation of Laboratory Animal Care International (AAALAC) approved the animal laboratory facility.

**Figure 1:**
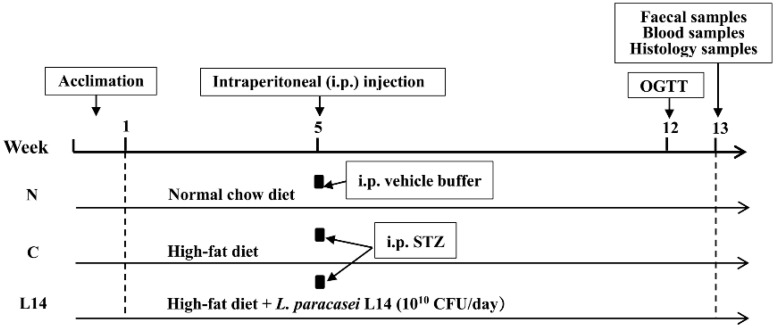
Experimental design. The T2DM rat model was established via HFD (induced IR) and low-dose STZ injection (specifically destroyed pancreatic β-cells). Therefore, insufficient insulin production after the STZ depletion of the β-cells failed to compensate for IR, resulting in hyperglycemia. N: normal group consisting of the control subjects without HFD/STZ. C: T2DM model subjects exposed to HFD/STZ. L14: the prevention group consisting of rats intragastrically administered with the *L. paracasei* L14 from weeks 1 to 12 and exposed to HFD/STZ treatment. The FBG levels, BW, and water and food consumption were recorded weekly. OGTT: oral glucose tolerance test.

The design of the animal experiments followed previously described methods [10], as shown in Figure 1. The experiments consisted of three groups (each with *n* = 6, 2 in each cage): the normal (N) group, diabetic control (C) group, and *L. paracasei* L14 (L14) protection group. After a 1-week acclimation period (week 0), the rats in group N received a normal chow between weeks 1 and 12, while the remaining animals received a HFD comprising 0.5% sodium cholate and 10% lard, 20% sucrose, and 67% of the normal diet. Meanwhile, the rats in the L14 group were orally administered with 10^10^ CFU/head of strain L14 once daily from weeks 1 to 12. At week 5, the rats in the C and L14 groups were subjected to a 16 h fasting period, after which they were intraperitoneally injected with fresh streptozotocin (STZ) (a cold 0.1 mol/L citrate buffer was used for dissolution at pH 4.2) at a 30 mg kg^–1^ body weight (BW) dosage, while the rats in the N were administered with an equal volume of vehicle buffer. One week after injection (at week 6), the fasting blood glucose (FBG) levels were recorded, while T2DM was deemed present at FBG > 11.1 mmol L^–1^.

### 2.2. The FBG, BW, and Water and Food Consumption

Blood was collected weekly from the tail veins of the rats to monitor the FBG, after which a glucometer was used for analysis (Roche Diagnostics, Mannheim, Germany). The BW and consumption of water and feed were also monitored every week.

### 2.3. Oral Glucose Tolerance Test (OGTT)

The OGTT was conducted at week 12. The rats were exposed to a fasting period of 12 h before receiving glucose at a dosage of 2 g kg^–1^ BW. The glucose levels in the blood were detected at 0 min, 30 min, 60 min, and 120 min.

### 2.4. Biochemical Parameters 

After 12 weeks, the rats were fasted for a 12 h period and sacrificed using isoflurane. Blood was acquired via the inferior vena cava and subjected to centrifugation to separate the serum for biochemical examination. The homeostatic model (HOMA-IR) was used to quantify the IR via the following calculation: HOMA-IR = FBG (mmol L^–1^) × Fasting blood insulin (mU L^–1^)/22.5. The insulin, lipid profiles, inflammatory cytokines, and oxidative stress were determined with commercial kits (Beijing Sino-UK Institute of Biological Technology, Beijing, China).

### 2.5. Histopathological Examination of the Pancreas and Liver Tissues

At the end of week 12, the pancreas and liver tissues isolated from the rats in the different groups were fixed in a 10% paraformaldehyde solution. Next, the paraffin was used to embed the fixed tissues, which were sliced into sections of 5 μm and stained with hematoxylin and eosin (HE) for examination via light microscopy.

### 2.6. Gut Microbiota Analysis

At the end of week 12, fresh feces were collected from the rats, and genomic DNA was extracted for the gut microbiota analyses, as previously described [11]. The extracted DNA was processed by an external service using an Illumina MiSeq Platform (Majorbio Bio-Pharm Technology, Shanghai, China). Furthermore, the amplification of the hypervariable V3-V4 area of the ribosomal 16S RNA gene was achieved using universal primers (338F: 5′-ACTCCTACGGGAGGCAGCAG-3′ and 806R: 5′-GGACTACHVGGGTWTCTAAT-3′). The Illumina MiSeq platform was used to perform further paired-end sequencing. Raw 16S rRNA reads have been made available on the SRA under the BioProject number PRJNA830649. After demultiplexing, the resulting sequences were quality filtered with fastp (v0.19.6) and merged with FLASH (v1.2.7). The optimized sequences were further de-noised using the DADA2 plugin in the QIIME2 (v.2020.2), and the de-noised sequences are usually called amplicon sequence variants (ASVs). To minimize the influence of sequencing depth on the subsequent analysis of alpha and beta diversity, the number of sequences from all samples was rarefied to 17,999, which still reached an average Good’s coverage of 99.98%. Taxonomic allocation of ASVs was performed using the classify-sklearn (Naive Bayes) implemented in QIIME2 and the SILVA 16S rRNA database (v138).

Bioinformatic analysis of the gut microbiota was carried out using the Majorbio Cloud platform (https://cloud.majorbio.com, accessed on 18 June 2023). Based on the ASVs information, alpha diversity indices, including Chao1 richness and Shannon index, were calculated with Mothur v1.30.2, while the Bray–Curtis dissimilarity Vegan v2.4.3 package was employed to determine the beta diversity via principal coordinate analysis (PCoA). Linear discriminant effect size (LEfSe) evaluation determined the differentially abundant biomarkers in the groups. The correlations between the T2DM-related indices and the top 28 most abundant genera were assessed using a pairwise Spearman’s rank correlation coefficient, which was presented in a heatmap.

### 2.7. Whole-Genome Sequencing of L. paracasei L14

The whole genome was sequenced using a combination of Illumina Novaseq6000 and Nanopore PromethION platforms by Shanghai Majorbio Bio-pharm Technology Co., Ltd. The Nanopore PromethION platform provides long-read sequences that are highly beneficial for the de novo assembly of genomes, especially for resolving complex regions; however, it has a relatively high error rate. On the other hand, the Illumina NovaSeq6000 platform only generates short-read sequences but is extremely accurate. Thus, the two platforms were combined to leverage the advantages of both platforms to achieve high-quality genome assembly with both long-distance continuity and high accuracy. The generated data were performed bioinformatics analysis using the free online platform of Majorbio Cloud Platform (http://cloud.majorbio.com, accessed on 10 June 2023). Specifically, the raw Illumina sequencing reads were quality-filtered using fastp v0.23.0. Nanopore reads were extracted, basecalled, demultiplexed, and trimmed using ONT Guppy with the minimum Q score cutoff of 7. The clean short and long reads were then co-assembled to construct complete genomes using Unicycle v0.4.8 [12], and Pilon v1.22 was then used to polish the assembly to reduce the rate of small errors. The coding sequences (CDS) were predicted using Prodigal v2.6.3 and annotated from NR, Swiss-Prot, Pfam, GO, COG, and KEGG databases using sequence alignment tools such as BLAST, Diamond, and HMMER. A whole-genome blast search (e-value < 10^−5^) was performed against the above databases.

### 2.8. Statistical Analysis

Data are shown as mean ± SEM. Statistical analyses were evaluated using the Student’s *t*-test when two groups were compared and one-way analysis of variance (ANOVA) followed by Duncan’s multiple comparison test when more than two groups were compared by SPSS v.20. A difference was deemed significant at *p* < 0.05.

## 3. Results and Discussion

### 3.1. L. paracasei L14 Reduces Hyperglycemia, Improves IR, and Protects Pancreatic Beta-Cell Function in the T2DM Rat Model Induced by HFD/STZ

**Figure 2:**
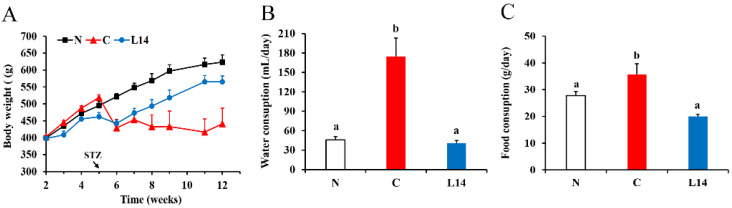
The effect of strain L14 on BW (**A**), water consumption (**B**), and food consumption (**C**). The data are shown as mean ± SEM (*n* = 6). Different letters signify substantially different values (*p <* 0.05).

The T2DM phenotypical traits such as weight loss, higher water consumption, as well as higher food intake were observed in the C group (Figure 2A–C). However, in group L14, there was only a slight drop in body weight after the STZ injection, followed by a steady rise. The average water and food intake were significantly lower in group L14 compared to group C levels (*p* < 0.05).

**Figure 3:**
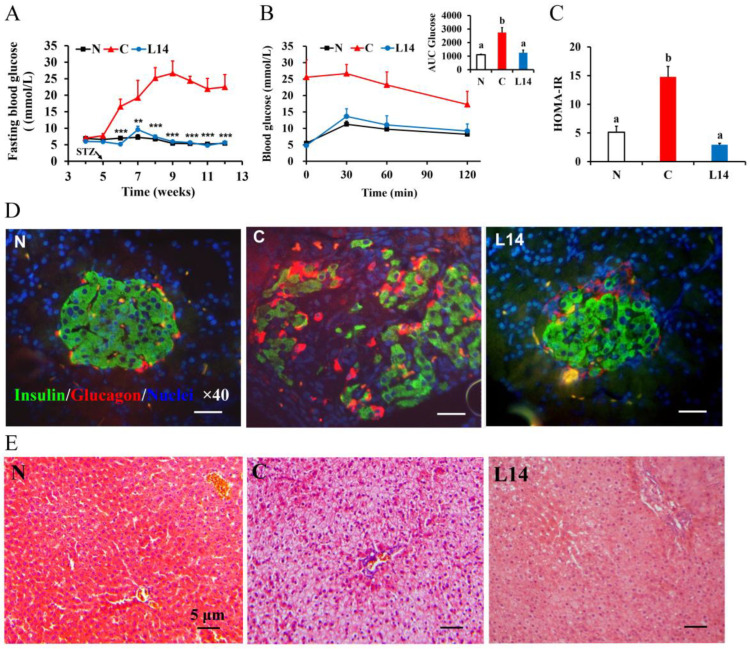
The effect of strain L14 on FBG (**A**), oral glucose tolerance at week 12 (**B**), and HOMA-IR (**C**). The data are shown as mean ± SEM (*n* = 6), *** *p* < 0.001 and ** *p* < 0.01. Different letters signify substantially different values (*p <* 0.05). (**D**) Representative islets after double-immunofluorescence staining, insulin (expressed in green), glucagon (expressed in red), and DAPI (expressed in blue) (scale bar = 10 µm and magnification ×400). (**E**) Representative liver samples after HE staining (scale bar = 5 µm and magnification ×100). N: normal group rats without HFD/STZ. C: T2DM model rats exposed to HFD/STZ. L14: the prevention group consisting of rats intragastrically administered with the *L. paracasei* L14 strain.

After STZ injection, the average FBG concentration of group C increased dramatically to 16.6 mmol L^–1^ in one week (Figure 3A, *p* < 0.05). This level was maintained until the conclusion of the experiment, indicating the success of the model establishment. Contrarily, the FBG in the L14 prevention group showed no obvious changes following STZ injection, which was markedly lower than that in group C, possibly because preventive *Lactobacillus* supplementation for four weeks before modeling provided some protection against diabetes.

This research also assessed the impact of strain L14 on glucose intolerance and IR. As shown in Figure 3B, the glucose tolerance in group C was severely impaired, displaying a significantly higher glucose area under the curve (AUC) after OGTT compared to the N group (*p* < 0.05). Preventive supplementation with strain L14 significantly reduced these levels by 54% compared to the C group (*p* < 0.05), exhibiting no substantial differences from group N (*p* > 0.05). The HOMA-IR in group C was considerably higher than in group N, indicating severe IR in group C (Figure 3C), while ingestion of the strain L14 significantly reduced the HOMA-IR level compared to group C (*p* < 0.05). This indicated that *L. paracasei* L14 improved the glucose tolerance and IR of the rats as a preventative measure.

The pancreatic histological analysis exposed obvious differences in the islet morphology (Figure 3D), as indicated via insulin and glucagon double-immunofluorescence staining. In group C, the number of β-cells (insulin-positive) in the islet was obviously decreased, and its arrangement was loose. Degenerative changes of β-cells, such as irregular nuclear size and morphology, nuclear shrinkage or karyolysis, and cell necrosis, were also found in group C. In addition, the number of α-cells (glucagon-positive) was considerably higher compared with the N group. However, the L14 group improved the pathological appearance with a marked increase in the β-cell number, which is closely arranged and evenly distributed in the islets. These results indicated that the strain L14 protected against β-cell destruction and restricted α-cell expansion in HFD/STZ-induced rats, consequently protecting β-cell function, which was in line with other findings that probiotics protect the islets of Langerhans from destruction and prevent hyperglycemia [10]. The administration of the L14 strain also significantly ameliorated the pathological state of the liver by improving its histological morphology (Figure 3E).

Recently, there have been many studies on regulating blood glucose by probiotics [13], but very few strains can restore blood glucose to normal levels. Interestingly, preventive supplementation of probiotic strain L14 was able to restore most of the biochemical parameters to normal levels, suggesting excellent effects for preventing or delaying the onset of T2DM. The ability to survive and colonize the gut is an important property for the probiotics to perform their beneficial functions. Thus, the gut colonization ability of strain L14 was examined, and results showed that it displayed an excellent colonization ability, with bacterial counts of 2.4 × 10^5^ CFU/g ileum and 2.9 × 10^6^ CFU/g colon, respectively.

### 3.2. L. paracasei L14 Ameliorates the Hyperlipidemia Status and Improves the Inflammatory and Antioxidant Status of the T2DM Rats Exposed to HFD/STZ 

**Figure 4:**
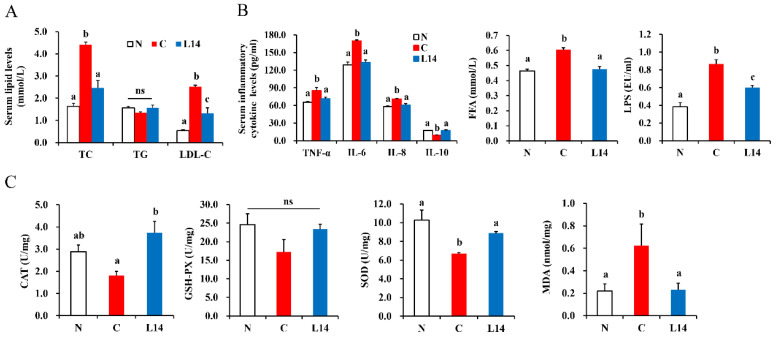
The effect of strain L14 on the biochemical parameters of diabetic rats. (**A**) Serum lipidlevels. (**B**) Serum inflammatory cytokines, FFA and LPS levels. (**C**) Oxidative stress levels. The data are shown as mean ± SEM (*n* = 6). Different letters signify substantially different values (*p* < 0.05). ns: *p* > 0.05.

T2DM is often accompanied by lipid metabolic disorders. In this study, the TC and LDL-C levels were considerably higher in group C than in the N group, while the increase in the two biochemical parameters of group L14 was substantially attenuated (Figure 4A, *p* < 0.05). An HFD causes inflammation, which is reportedly the primary factor contributing to IR [14]. Therefore, this study investigated whether the strain L14 could alleviate inflammation in diabetic rats. As shown in Figure 4B, the TNF-α, IL-6, and IL-8 pro-inflammatory factors in group C were substantially higher than in the N group (*p* < 0.05), while that of the anti-inflammatory factor, IL-10, displayed a considerable decline. However, in the L14 intervention group, the pro-inflammatory factors were downregulated, and the IL-10 level was upregulated. Studies have shown that FFA and LPS can trigger pro-inflammatory factor production and inhibit the insulin signaling pathway, consequently aggravating IR [15,16,17]. The C group displayed substantially higher FFA and LPS levels than the N group. However, supplementation with strain L14 significantly reduced the FFA and LPS levels (Figure 4B, *p* < 0.05). These results indicated that *L. paracasei* strain could inhibit the inflammatory response by regulating FFA, LPS, and inflammation-related cytokine levels to alleviate IR in the HFD/STZ subjects.

Studies have shown that oxidative stress is a vital trigger for IR, as well as the subsequent occurrence, development, and complications of T2DM [18]. Oxidative stress also contributes to other disorders, including aging, cancer, atherosclerosis, and neurodegenerative disease [19]. Figure 4C shows the effect of *Lactobacillus* on oxidative stress in rat livers. The levels of the CAT, GSH-PX, and SOD antioxidant enzymes in the C group rats were considerably lower than in group N (*p* < 0.05). The MDA content was substantially higher, suggesting impaired antioxidant capacity in the C group. The administration of *L. paracasei* L14 substantially elevated the CAT, GSH-PX, and SOD content, while the MDA decreased, even returning to normal levels. Therefore, *L. paracasei* improved oxidative stress levels in T2DM rats. Consequently, it is speculated that the *L. paracasei* L14 strain may protect the host from oxidative damage caused by hyperglycemia and hyperlipidemia via its antioxidant effect.

### 3.3. L. paracasei L14 Modulates the Intestinal Microbiota to Alleviate HFD/STZ-Induced Dysbiosis

**Figure 5:**
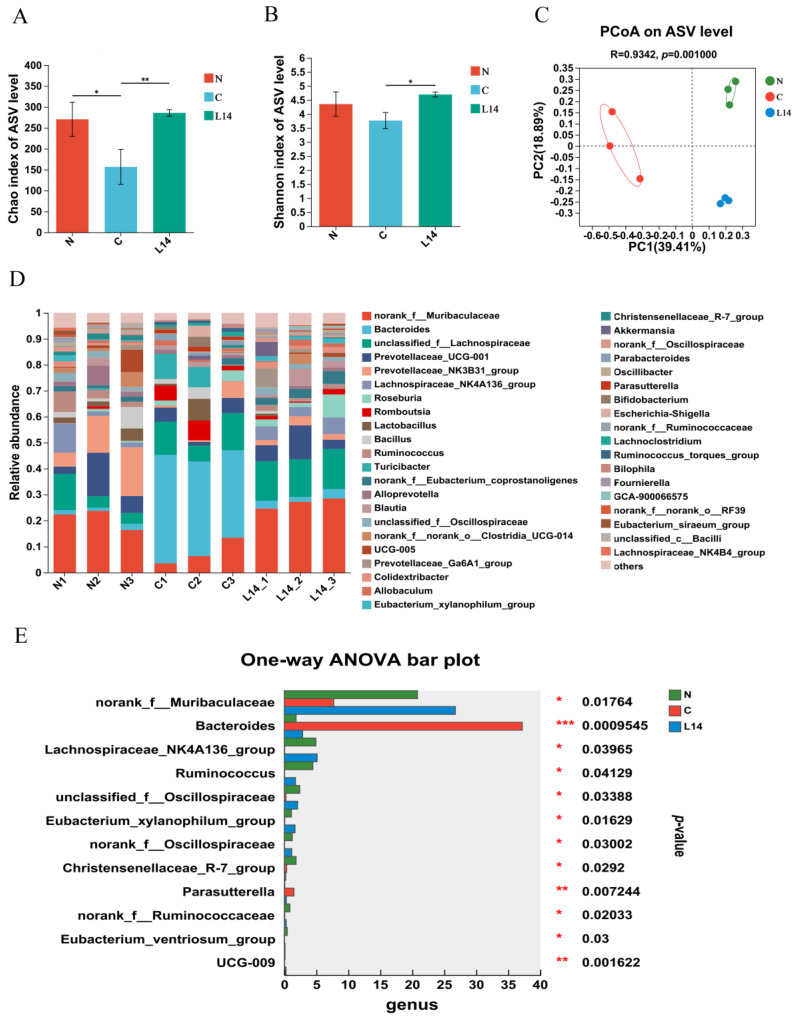
The strain L14 modulated the gut microbiota in T2DM rats. (**A**) The α-diversity of the microbiota in the feces. Data are shown as mean ± SEM (*n* = 3), while different letters indicate substantially different values (*p <* 0.05). (**B**) The PCoA based on the Bray–Curtis distance. (**C**) The average relative gut microbiota abundance of the samples at a genus level. (**D**) The proportional abundance of the bacteria displays the most significant changes at the genus level. (**E**) The proportional abundance of the bacteria displaying the most significant changes at the genus level, * *p* <0.05, ** *p* < 0.01 and *** *p* < 0.001.

The gut microbiome is vital for the pathophysiology of T2DM, influencing gut barrier function, systemic inflammation, and IR [20,21]. The modulation of the microbiota is also increasingly accepted for managing this disease. This study examined whether gut microbiota modification was responsible for the enhanced effect of L14 on T2DM. First, the α-diversity metric was measured using the Ace and Shannon indexes, respectively, to evaluate microbial richness and diversity (Figure 5A,B). The Ace and Shannon indexes in the L14 group were substantially higher than the C group (*p* < 0.05), indicating an increase in the number of species and diversity after L14 treatment. PCoA based on the Bray–Curtis distance depicted the dissimilarities between the fecal microbiota of the different groups (*p* = 0.001, Figure 5C).

HFD/STZ treatment also affected the intestinal bacterial composition of the rats. Oral gavage with strain L14 significantly changed the genus level proportion compared to group C (Figure 5D). The *Bacteroides*, *Christensenellaceae*_R-7_group, and *Parasutterella* levels decreased significantly, while that of norank_f_*Muribaculaceae*, *Lachnospiraceae*_NK4A136_group, *Ruminococcus*, unclassified_f_*Oscillospiraceae*, and *Eubacterium_xylanophilum*_group were considerably higher in the HFD/STZ-treated rats of the L14 group (Figure 5E). The results indicated that the L14 strain ameliorated HFD/STZ-induced dysbiosis by regulating the intestinal microbiota structure associated with α- and β-diversity and the proportion of potentially beneficial species.

**Figure 6:**
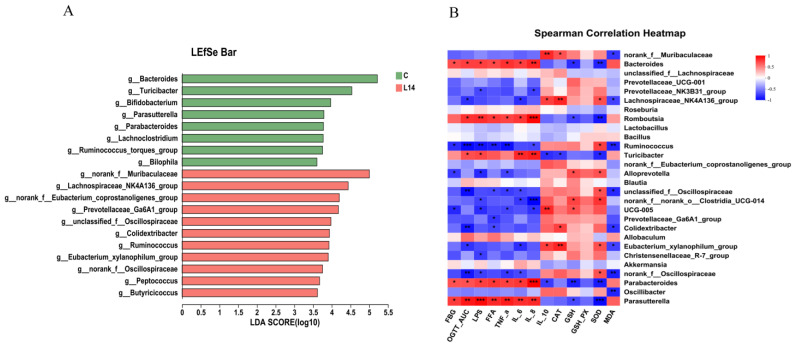
The LEfSe results of the significantly different biomarkers between the C and L14 groups according to the LDA score (log 10) (**A**) and Spearman correlation heatmap of the top 28 genera and T2DM-related parameters (**B**). The colors signify negative correlations (blue) and positive correlations (red). Substantial correlations are expressed as * *p* < 0.05, ** *p* < 0.01, and *** *p* < 0.001.

To further determine the differential biomarkers between the C and L14 groups, the LEfSe method was used to calculate the LDA scores. Results indicated that the L14 group was enriched with potential beneficial microbiota, such as norank_f_*Muribaculaceae* and *Lachnospiraceae*_NK4A136_group (Figure 6A). *Muribaculaceae* was reported to show a significant negative correlation with inflammatory status [22,23]. The *Lachnospiraceae*_NK4A136_group was associated with increased butyric or propionic acid production, as well as increased gut hormone secretion and decreased FBG, pro-inflammatory factors, and lipid profiles [11,24]. Contrarily, *Bacteroides*, *Turicibacter,* and *[Ruminococcus]_torques*_group were enriched in the C group (Figure 6A). *Bacteroides* (Gram-negative bacteria) are strongly associated with a long-term fat diet and the impairment of intestinal barrier function. This leads to a higher bacterial LPS translocation into the blood, inducing low-grade inflammation and decreasing insulin sensitivity [25]. *Turicibacter* and *[Ruminococcus]_torques*_group are positively correlated with colitis, IR, and diabetes by affecting gut health [26]. Significantly increased levels of these bacteria were found during the early diabetic stage [27].

The interaction between the microbiome and T2DM-related parameters was illustrated using a Spearman correlation heatmap (Figure 6B). The species enriched in the L14 group, namely norank_f_*Muribaculaceae*, and *Lachnospiraceae*_NK4A136_group, were positively associated with IL-10 and CAT levels and negatively linked to the MDA level. Moreover, the *Prevotellaceae*_NK3B31_group and *Ruminococcus* displayed a negative correlation with LPS, IL-8, and MDA levels and a positive correlation with SOD levels. The *Alloprevotella* and *Eubacterium_xylanophilum*_group were negatively associated with LPS, IL-6, and TNF-α and positively associated with SOD and CAT levels. Contrarily, the bacteria in the C group, namely *Bacteroides*, *Romboutsia,* and *Turicibacter*, were positively associated with the FBG, OGTT_AUC, LPS, FFA, TNF-α, IL-6, and IL-8 levels, and negatively correlated with GSH and SOD. Therefore, altered gut microbiota homeostasis affects the development of T2DM, and supplementation with the probiotic strain L14 partially restores the gut microbiota to relieve oxidative stress, inflammation, and IR.

It would have been better to include the initial gut microbiota data in this experiment; however, in this study, exactly consistent experimental conditions were controlled at the first-week acclimation period, the rats were randomly divided, and the L14 group received strain L14 administration since week two. Therefore, it could exclude the possibility that differential gut microbiota in the L14 group come from the beginning.

### 3.4. Whole-Genome Analysis of L. paracasei L14

**Figure 7:**
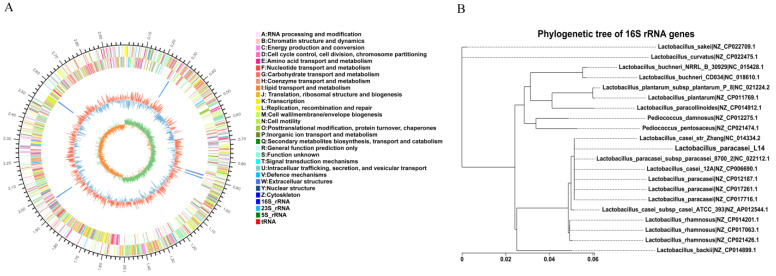
Whole-genome sequencing of *L. paracasei* L14. (**A**) Circular genome map of *L. paracasei* L14 created by software Circos. From outside to the center: (1) Genome size identification. (2) Coding sequences on forward strain (color by COG categories). (3) Coding sequences on reverse strand (color by COG categories). (4) rRNA and tRNA. (5) GC content. (6) GC skew (green and orange). (**B**) 16s rRNA gene-based phylogenetic tree.

To better understand the molecular basis of the probiotic activities of strain L14, whole-genome sequencing was performed (Figure 7A). It contains a circular chromosome and a plasmid of a total of 3,003,403 bp and (C + G)% 46.35%. The chromosome contains 2999 protein-coding sequences (CDS), 59 tRNA, and 15 rRNA genes. Phylogenetic tree of 16S rRNA gene revealed that L14 was 100% identical to *L. casei* Zhang, *L. paracasei* 8700, *L. casei* 12A, *L. paracasei* CAUH 35 (CP012187.1), *L. paracasei* FAM18149 (CP017261.1), and *L. paracasei* TK1501 (CP017716.1) (Figure 7B). The comparative genome analysis of the above strains showed that the genome size ranged from 2.71 to 2.94 Mb, with the same GC content being 46% (Table 1). The strains contained one to six chromosomes, and the number of protein-coding genes ranged from 2641 to 2803.

According to the genome annotation, 16 antioxidant-related genes (Table 2) were found. The oxidoreductase-related gene clusters mainly include *ahpC*: Alkyl hydroperoxide reductase C, *gpx*: Glutathione peroxidase, *nrdH*: Glutaredoxin-like protein, Mn*SOD*: Superoxide dismutase [Mn], *mntH*: Nramp family divalent metal transporter, *trxA*: Thioredoxin, *trxB*: Thioredoxin reductase, *msrA*: peptide-methionine (S)-S-oxide reductase, *msrB*: peptide-methionine (R)-S-oxide reductase, NAD(P)/FAD-dependent oxidoreductase, and *srlD*: SDR family oxidoreductase. Among these genes, *ahpC*, *trxA*, *trxB*, and *msrAB* constitute the thioredoxin (Trxs) system, and gpx and *nrdH* constitute the glutathione-glutaredoxin (Grxs) system. The Trxs and Grxs play an essential role in protecting cells from ROS damage by controlling the thiol–disulfide balance [28]. Superoxide dismutase [Mn] (Mn*SOD*) and Glutathione peroxidase (*gpx*) are among the most important antioxidant enzymes preventing cells from oxidative stress [29]. Therefore, it was inferred that these antioxidant-related genes were responsible for the excellent antioxidant capacity of *L. paracasei* L14 in vivo.

In addition, one EPS cluster composed of 20 genes (from LPAR_2145 to LPAR_2164) was found in the genome (Table 3). Our previous experiments also found that this strain could form ropy colonies on MRS plates, and a long string could produce when the colony was picked with pipette tips. These indicated that strain L14 possesses better EPS production ability. EPS are important microbial metabolites that show a significant influence on bacterial adhesion, biofilm formation, aggregation, and survival [30]. In addition to being widely used in improving the quality of food products, probiotics-derived EPS have been recently reported to have various health-promoting effects such as antioxidant, anti-diabetic, immunomodulatory, antimicrobial, and antitumor activity [31]. Therefore, the good probiotic properties of strain L14 might be partly attributed to its production of EPS. At the same time, EPS could be extracted to exploit novel value-added functional foods or be used in the pharmaceutical industry.

## 4. Conclusions

Preventative supplementation of probiotic *L. paracasei* L14 displays an excellent preventive effect on T2DM rats by improving oxidative stress, IR, inflammation, dyslipidemia, and protecting beta-cell and liver function while modulating the intestinal microbiota to alleviate gut dysbiosis. Whole-genome sequencing of *L. paracasei* L14 found some important genes associated with the antioxidant system and EPS biosynthesis, providing a critical molecular basis for its probiotic properties. Thus, probiotic *L. paracasei* L14 has good potential for applications in food adjuvants or pharmaceutical industries to prevent the onset of diabetes. Further clinical trials are needed to validate the efficacy of *L. paracasei* L14 in humans.

## Figures and Tables

**Table 1:** Comparison of the genome of *L. paracasei* L14 with the genomes of similar strains.

No.	Organism	Strain	Assembly Accession	Genome Size (Mb)	Scaffold No.	Chromosome No.	G + C (%)	Protein-Coding Sequences
1	*L. paracasei*	Zhang	GCF_000019245.4	2.86	2	2	46	2641
2	*L. paracasei*	8700	GCF_000155515.2	2.94	3	3	46	2803
3	*L. casei*	12A	GCF_000359565.2	2.91	1	1	46	2682
4	*L. paracasei*	CAUH35	GCF_001191565.1	2.77	5	5	46	2690
5	*L. paracasei*	FAM18149	GCF_002442835.1	2.71	6	6	46	2730
6	*L. paracasei*	TK1501	GCF_002257625.1	2.94	1	1	46.5	2665

**Table 2:** Antioxidant-related genes in the *L. paracasei* L14 genome.

Locus Tag(s)	Accession No.	Gene	Predicted Encoded Function	Protein Size
PUK88_12395	WP003567571.1	*ahpC*	Alkyl hydroperoxide reductase C	187aa
PUK88_04685	WP003601666.1	*gpx*	Glutathione peroxidase	157aa
PUK88_07930	WP005685874.1	*nrdH*	Glutaredoxin-like protein	76aa
PUK88_09810	WP012491719.1		Superoxide dismutase [Mn]	205aa
PUK88_00185	WP011673942.1	*mntH*	Nramp family divalent metal transporter	458aa
PUK88_04200	WP010493758.1	*trxA*	Thioredoxin	103aa
PUK88_01345	WP003568841.1		Thioredoxin family protein	104aa
PUK88_04960	WP003564228.1	*trxB*	Thioredoxin–disulfide reductase	315aa
PUK88_06640	WP003590482.1	*msrA*	peptide–methionine (S)-S-oxide reductase	276aa
PUK88_08180	WP003565681.1	*msrB*	peptide–methionine (R)-S-oxide reductase	150aa
PUK88_06850	WP003574830.1		GAF domain-containing protein	160aa
PUK88_11715	WP003595937.1		NAD(P)/FAD-dependent oxidoreductase	632aa
PUK88_02000	WP016370227.1		sorbitol-6-phosphate dehydrogenase subunit	266aa
PUK88_02065	WP019862015.1	*srlD*	SDR family oxidoreductase	267aa
PUK88_02200	WP003577685.1		sorbitol-6-phosphate dehydrogenase subunit	266aa
PUK88_13525	WP019862015.1		SDR family oxidoreductase	266aa

**Table 3:** Putative EPS cluster in *L. paracasei* L14.

Locus Tag(s)	Predicted Encoded Function	Protein Size	Domain
PUK88_10270	polysaccharide deacetylase	332aa	Polysaccharide deacetylase (PF01522)
PUK88_10275	sulfatase-like hydrolase/transferase	774aa	Sulfatase (PF00884)
PUK88_10280	EpsG family protein	349aa	EpsG family (PF14897)
PUK88_10285	CpsD/CapB family tyrosine–protein kinase	211aa	AAA domain (PF13614); NUBPL iron-transfer P-loop NTPase (PF10609); CobQ/CobB/MinD/ParA nucleotide-binding domain (PF01656)
PUK88_10290	Wzz/FepE/Etk N-terminal domain-containing protein	230aa	Chain-length-determinant protein (PF02706)
PUK88_10295	O-unit flippase-like protein	479aa	-
PUK88_10300	glycosyltransferase	378aa	Glycosyl transferase group 1 (PF00534)
PUK88_10305	glycosyltransferase family 2 protein	321aa	Glycosyl transferase family 2 (PF00535); Glycosyltransferase-like family 2 (PF13641); Glycosyltransferase-like family 2 (PF10111)
PUK88_10310	hypothetical protein	558aa	-
PUK88_10315	hypothetical protein	579aa	-
PUK88_10320	GW dipeptide domain-containing protein	693aa	GW (Gly-Tryp) dipeptide domain (PF01183); Glycosyl hydrolases family 25 (PF13457)
PUK88_10325	glycosyltransferase family 39 protein	495aa	Dolichyl-phosphate-mannose-protein mannosyltransferase (PF13231)
PUK88_10330	acyltransferase	346aa	Acyltransferase family (PF01757)
PUK88_10335	glycosyltransferase family 2 protein	330aa	Glycosyl transferase family 2 (PF00535)
PUK88_10340	UDP-N-acetylglucosamine 2-epimerase, wecB	380aa	UDP-N-acetylglucosamine 2-epimerase (PF02350)
PUK88_10345	hypothetical protein	288aa	-
PUK88_10350	sugar transferase	466aa	Bacterial sugar transferase (PF13727); CoA-binding domain (PF02397)
PUK88_10355	Glycosyl transferase	246aa	Glycosyltransferase sugar-binding region containing DXD motif (PF04488)
PUK88_10360	glycosyltransferase family 2 protein	252aa	Glycosyl transferase family 2 (PF00535)

## Data Availability

The complete genome sequence of *L. paracasei* L14 was deposited in the GenBank (CP129524, https://www.ncbi.nlm.nih.gov/nuccore/CP129524.1/, accessed on 26 June 2023). This strain has been deposited in the China General Microbiological Culture Collection Center (CGMCC No. 4015).
